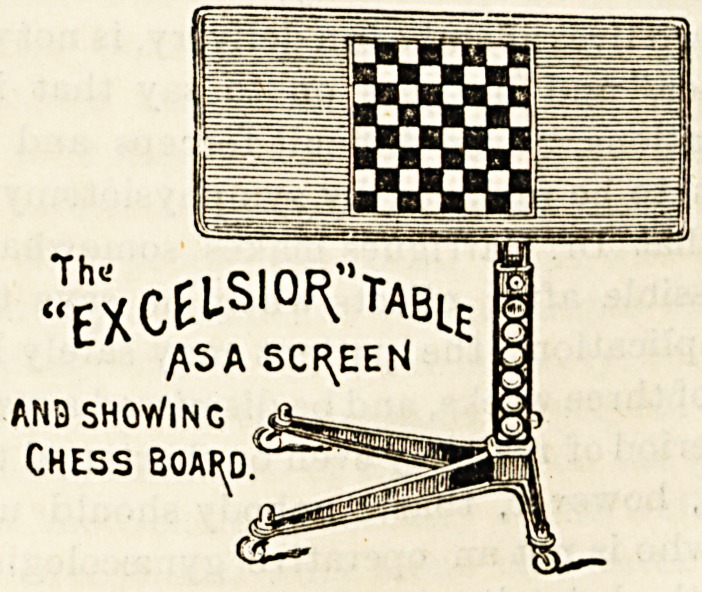# New Appliances and Things Medical

**Published:** 1903-01-31

**Authors:** 


### NEW APPLIANCES AND THINGS MEDICAL.

**[We shall be glad to receive at our Office, 28 & 29 Southampton Street, Strand, London, W.C., from the manufacturers, specimens of all new preparations and appliances which may be brought out from time to time.]**

### THE EXCELSIOR TABLE.

(THE WINCYCLE TRADING CO., LIM., 106 GREAT SAFFRON HILL, LONDON, E.C.)

THE Excelsior bed-table is one of those aids to comfort which, when once utilised, cause one to wonder how one had managed without it previously. We use the word bed-table, but although such a table is quite indispensable to the comfort of the invalid, the able-bodied will find it equally convenient owing to its adaptability. It forms an admirable reading desk, writing table, or fire screen for the use of convalescents. For the invalid, it renders meals in the bedroom comfortable and mitigates fatigue. It renders writing in bed not only possible but perfectly convenient, as the table can be adjusted to any angle to suit the recumbent invalid. If a pencil is used, the invalid can write without raising the head at all. When not required, it can be easily pushed away from the bed by the patient without assistance, and as it folds down it occupies little room. It is an excellent table at the moderate price of 35s., and doctors, nurses, and institutions can obtain it at a reduction from the makers.

## Figures and Tables

**Figure f1:**
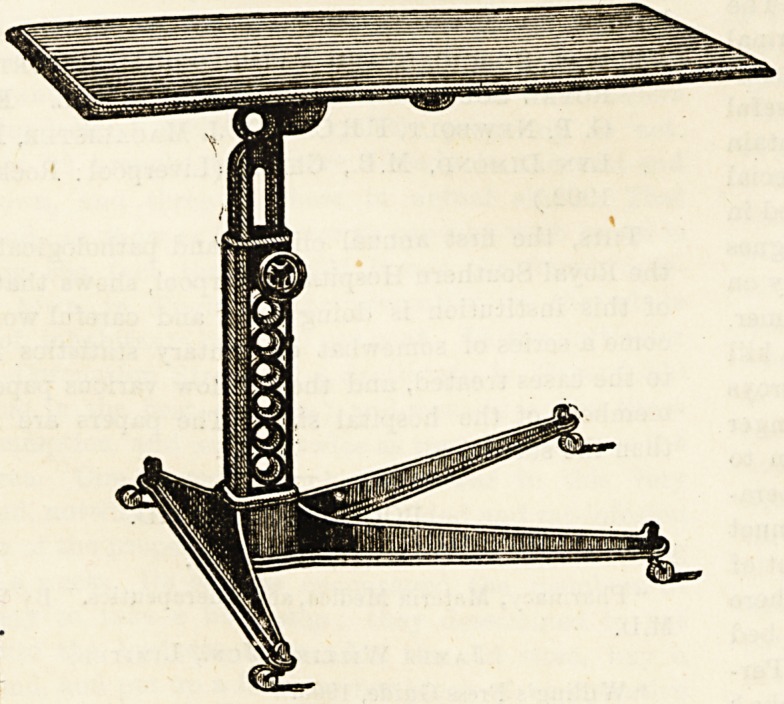


**Figure f2:**
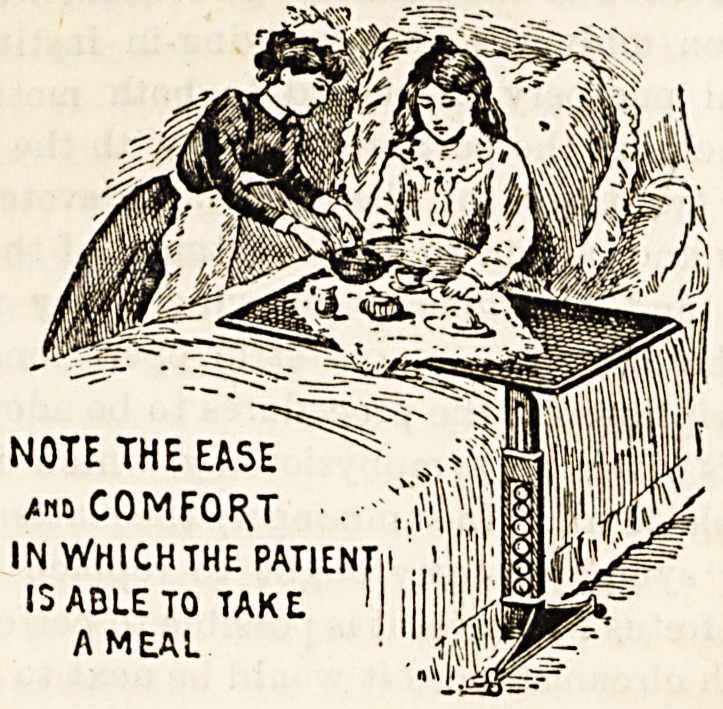


**Figure f3:**